# Which has more complications?—Shockwave lithotripsy versus endoscopic treatment of renal calculi with 1‐year follow‐up in an Australian population

**DOI:** 10.1002/bco2.71

**Published:** 2021-07-01

**Authors:** Matthew Farag, Gregory S. Jack, Nathan Papa, Lih‐Ming Wong, Damien M. Bolton, Daniel Lenaghan

**Affiliations:** ^1^ Department of Urology Austin Health, University of Melbourne Heidelberg VIC Australia; ^2^ Department of Surgery St Vincent's Hospital Melbourne Fitzroy Australia

**Keywords:** intra‐renal pressure, pyeloscopy, pyelovenous‐backflow, retrograde intra‐renal surgery, RIRS, sepsis, ureteropyeloscopy

## Abstract

**Introduction and objectives:**

Renal calculi are a common medical problem with incidence rates calculated to be approximately 6%‐9% in men & 3%‐4% in women worldwide. Incidence appears to be increasing. This study compares emergency presentations and unplanned readmissions between extracorporeal shock wave lithotripsy (SWL) and pyeloscopic stone treatment in the population of Victoria, Australia after 1‐year follow‐up.

**Methods:**

This is a population study comparing all patients with renal calculi electively treated with SWL to those initially treated with flexible ureteroscopy (URS) in Victoria, Australia. We used data linkage across the state of Victoria to follow patients treated with either modality in a 12 months period (with no urological surgery in the prior 12 months). Each patient's emergency presentations and subsequent re‐admissions were followed up for 1 year after their index treatment to assess for stone complications. We assessed for selection bias between the two patient groups by comparing age, gender, insurance status, geographical location, and comorbidity scores.

**Results:**

We report stone‐related complications for 739 flexible URS and 1317 SWL procedures undertaken across public and private hospitals in Victoria over 12 months. Unplanned emergency presentations within 60‐days of surgery were (22/739) 2.98% for flexible URS patients and (83/1317) 6.30% for SWL patients (*P* = .001); however, at 12 months, this became 16.23% (120/739) for flexible URS patients and 12.83% (169/1317) for SWL patients (*P* = .034). Flexible URS patients were more likely than SWL patients to be admitted with 71.76% of flexible URS versus 53.97% of SWL patients requiring an admission at any given emergency presentation (*P* ≤ .001) within 12 months. On multivariate analysis, both flexible URS ([OR] 1.67, CI 1.23‐2.26, *P* = .001) and being a public patient ([OR] 3.06, CI 2.24‐4.18, *P* < .001) significantly increased the likelihood that patients required an unplanned re‐admission within 12 months.

**Conclusions:**

There is work needed to reduce emergency presentations and unplanned re‐admissions after both SWL and flexible URS. At 12‐months follow‐up, unplanned emergency visits and re‐admission rates were significantly more after flexible URS. Symptoms at emergency presentation indicate that better education regarding stent management is needed, especially in the public health care system.

## 1 INTRODUCTION

Renal Calculi are a common clinical presentation globally, with recorded prevalence varying between 1% and 20%.^1^ Australian department of health data reports that renal calculi contributed to 5% of all emergency presentations; furthermore, it has been observed that 50% of patients have at least one repeat episode and 10%‐20% can have three or more repeat episodes.^2^, ^3^ We sought to explore the surgical management of renal calculi, and in particular whether the choice of the procedure can affect the burden of this disease on the health care system.

Although the occurrence of renal calculi varies by age, race, and sex,^4^ the overall prevalence of renal calculi seems to be increasing,^2^, ^4^ where in the last 20 years alone increases more than 37% have been observed in some countries.^3^, ^5^, ^6^ Furthermore, it has been estimated that one's lifetime risk of developing a kidney stone is 8%‐10%.^7^ With the financial burden of kidney calculi estimated to cost $1.8‐2.4 billion annually in the United States,^8^, ^9^ together with the associated morbidity and loss of productivity to the patient,^10^ it is more important than ever clear and effective mechanisms for stone management are outlined. Interestingly, in Australia, studies have found that the rates of surgical intervention for stone treatment doubled between 1980 and 1997, despite only a 13% increase in first time ED presentations.^10^ This suggests stone surgery may be increasing at a rate greater than that attributable to symptomatic renal colic presentations. Additionally, flexible and rigid ureteroscopic (URS) lithotripsy is increasing in prevalence accompanied by relative and absolute reductions in both shock wave lithotripsy (SWL) and percutaneous nephrolithotomy (PCNL).^11^

It is widely known that in 90% of cases, calculi of ≤5 mm are likely to pass spontaneously without the need for medical or surgical intervention, with the pass rate reducing to 50% for calculi between 5 and 10 mm.^2^, ^12^ This information has guided the management for calculi, and according to the American Urological Association (AUA) guidelines, uncomplicated ureteral calculi of less than or equal to 10 mm should undergo observation, while medical management with alpha‐blockers can be considered for more distal calculi of similar size (Evidence level grade B).^13^ Surgical treatment can be considered if a period of observation for 4‐6 weeks has been unsuccessful (Evidence level grade C).^13^ Furthermore, it is widely accepted that larger calculi require more invasive procedures, with strong evidence for PCNL in the removal of renal calculi >20 mm.^1^, ^13^

In regards to renal/ureteric calculi in the intermediate size, the literature remains ambiguous in regards to the best treatment option. For these calculi, SWL and URS are two interventions that can be used in the definitive management. Historically, proximal ureteric calculi have been managed with SWL for calculi <10 mm, while URS is more indicated for calculi >10 mm.^14^ As per the AUA guidelines, URS is more effective at stone removal and provides patients with a higher stone‐free rate following a single procedure (evidence level grade B).^13^ URS is, therefore, recommended as the first‐line treatment for patients who are not suitable for medical or observational management, in patients with a mid or a distal ureteral stone (Evidence level grade B).^13^ In those patients where URS is not an option, SWL can be offered as the second line. However, SWL has lower complication rate and greater safety profile (Evidence level grade B).^13^

## 2 METHODS

### 2.1 Study design

This is a population study, in Victoria Australia, comparing two different treatment groups (SWL and Flexible URS) for the treatment of renal calculi (excluding ureteric calculi). We have obtained data from the Center for Victorian Data Linkage corresponding to every renal stone procedure undertaken in both private/public hospitals across Victoria from 1 January 2013 to 31 December 2013. Each patient included had an Index Stone Treatment (IST) with either SWL or flexible URS in 2013, with patients with renal stone treatment in the prior 12 months excluded. Data included patients from 145 public and private hospitals. Linked data for each study patient were then obtained and analyzed for 1 year post their IST to assess for stone complications, this database also captured any emergency presentation or hospital re‐admission in a 5‐year follow‐up period.

### 2.2 End points

Stone complications were defined as any unplanned stone‐related presentation to emergency or re‐admission with symptoms including renal colic, fever, and urinary tract infection.

### 2.3 Statistical analysis

Calculations were performed using Stata/MP version 13.0 for Mac (StataCorp LP). Variables were checked for skewness and kurtosis to determine normality. Clinical and demographic features are presented as medians [interquartile range] and means (± standard deviation) for non‐parametric and parametric data respectively. Differences between continuous parametric variables were examined with the *t* test; the Wilcoxon rank‐sum test or the Wilcoxon‐Mann‐Whitney test were used for non‐normally distributed continuous and ordinal variables, while differences between dichotomous variables were evaluated with the *χ* ^2^ test or the Fishers exact test (Tables 1 and 2). *P*‐values throughout the results were two‐sided. Logistic regression was performed on clinically and statistically significant variables as part of a multivariate analysis.

**TABLE 1:** Descriptive statistics

Number in cohort	Flexible ureteroscopy	ESWL	*P* value
	739	1317	
Median age	55 [47‐66]	54 [45‐64]	.1770
Gender (male) %	68%	69%	.3372
Private cases %	62%	56%	.0011
Ureteric stent inserted %	73%	3%	<.001
LOS (days) of initial procedure (Mean ± SD)	1.46 ± 2.74	1.06 ± 0.65	<.001
ICU admission post procedure %	0.54%	0.23%	.2591
ASA grade (median [IQR])	2 [1‐3]	2 [1‐3]	.8563

**TABLE 2:** Post‐operative unplanned emergency presentations

Emergency presentations	Flexible ureteroscopy *N* = 739	ESWL *N* = 1317	*P* value
Incidence at 60 days %	2.98%	6.30%	.0011
Incidence at 12 months %	16.23%	12.83%	.0341
Admission at first emergency presentation	66.41%	45.61%	.0010
Triage category at emergency presentation (median, IQR)	3 [2‐3]	3 [2‐3]	.7842
Patient stented at presentation	89%	3%	>.001
Length of stay in ED (mins)	210 [142‐306]	209 [120‐296]	.3602
Incidence of UTI/Urosepsis	2.44%	1.97%	.2841
Incidence of renal colic	13.80%	10.93%	.0562

## 3 RESULTS

A total of 2056 patients underwent either SWL or flexible URS for renal stone disease in 2013, and no preceding stone surgery in the preceding 12 months. Of these, 739 patients had undergone flexible URS and 1317 patients had undergone SWL at multiple public and private sites across Victoria, Australia. Patients were 18 years old and over and the median age at IST was similar in both groups (*P* = .137) (Table 1). Sixty eight percent of patients were male and 32% female, this was also similar between the two groups (*P* = .340). Seventy three percent of flexible URS patients had a ureteric stent inserted at the end of their procedure compared with only 3% of SWL patients (*P* = .001). Sixty two percent of flexible URS procedures and 56% of SWL procedures were completed in the private system (*P* = .001). The mean length of stay related to surgery was significantly longer for flexible URS, 1.46 ± 2.74 days ± SD versus 1.06 ± 0.65 days ± SD for SWL, (*P* < .001). (Table 1) Regardless of procedure type, patients treated initially in public were 2.17 times more likely to attend ED than those treated in private (*P* ≤ .001).

Unplanned emergency presentations within 60 days of IST were (22/739) 2.98% for flexible URS patients and (83/1317) 6.30% for SWL patients (*P* = .001) (Table 2). Of the flexible URS patients presenting to emergency, 89% had a ureteric stent in situ. At 12 months post‐IST, emergency presentations were 16.23% (120/739) for flexible URS patients and 12.83% (169/1317) for SWL patients (*P* = .034). These 289 emergency re‐presentations accounted for 40 flexible URS patients and 106 SWL patients, meaning SWL patients were likely to present 1.59 times/patient to emergency for stone‐related complications, compared to 3.00 times/patient for flexible URS (*P* = .012).

At their first emergency presentation post‐IST, 45.61% of SWL patients were admitted compared to 66.41% of flexible URS patients (*P* < .001). The discrepancy between the two cohorts remained at subsequent presentations to emergency with 71.76% of flexible URS versus 53.97% of SWL patients requiring an admission at any given emergency presentation (*P* ≤ .001). The emergency triage acuity/category assigned to each patient was on average statistically similar between the two cohorts, 59.54% of flexible URS patients and 56.07% SWL patients assigned a “Category 3” (*P* = .780).

There was no significant difference in the median time spent in the emergency department for both cohorts, SWL 209 [120‐296] minutes versus flexible URS 210 [142‐306] minutes (*P* = .360). Renal colic was the most common reason for emergency presentation, in 85.12% (246/289) of re‐presentations, 10.93% (144/1317) SWL versus verse 13.80% (102/739) flexible URS (*P* = .056). UTI was diagnosed in 15.22% (44/289) of representations, the incidence for SWL was 1.97% (44/1317) versus 2.44% (18/739) for flexible URS (*P* = .284).

We also analyzed total re‐admissions in the 12 months following IST, this rate was (873/739) 1.18 re‐admissions per procedure for flexible URS patients and (653/1317) 0.50 re‐admissions per procedure for SWL patients (*P* < .001). This rate included routine flexible cystoscopy and removal of ureteric stents; the average length of duration for a stent inserted was 17.26 days. We also analyzed unplanned re‐admissions within 12 months comprised a small number of these total re‐admissions, the rate was 11.64% (86/739) for flexible URS patients and 9.03% (119/1317) for SWL patients (*P* = .036) (Table 3). Furthermore, we looked at the length of stay for unplanned re‐admissions, for flexible URS (72/739) 9.74% of patients required an overnight stay compared to (92/1317) 7.00% of SWL patients *P* = .029. The average length of stay for unplanned re‐admissions for flexible URS patients was 4.41 days, confidence interval [3.24‐5.57] compared to 2.95 days, confidence interval [2.32‐3.58] for SWL patients *P* = .010.

**TABLE 3:** Post‐operative re‐admissions within 12 months

Readmissions (*N*)	Flexible Ureteroscopy (739)	ESWL (1317)	
Unplanned/planned readmissions per patient	1.18	0.54	<.001
Incidence of planned %	106.50%	40.55%	<.001
Incidence of unplanned %	11.64%	9.03%	.0363
Unplanned readmissions 28 days	5.17%	6.22%	.7429
Unplanned re‐admissions with LOS > 1	9.74%	7.00%	.0294
Average LOS (days) at unplanned readmission (Confidence Interval)	4.41 (3.24‐5.57)	2.95 (2.32‐3.58)	.0102
Proportion of unplanned readmissions requiring a procedure	7.55%	4.32%	<.001
ICU required during readmission %	0.6%	0%	.2599

**FIGURE 1:**
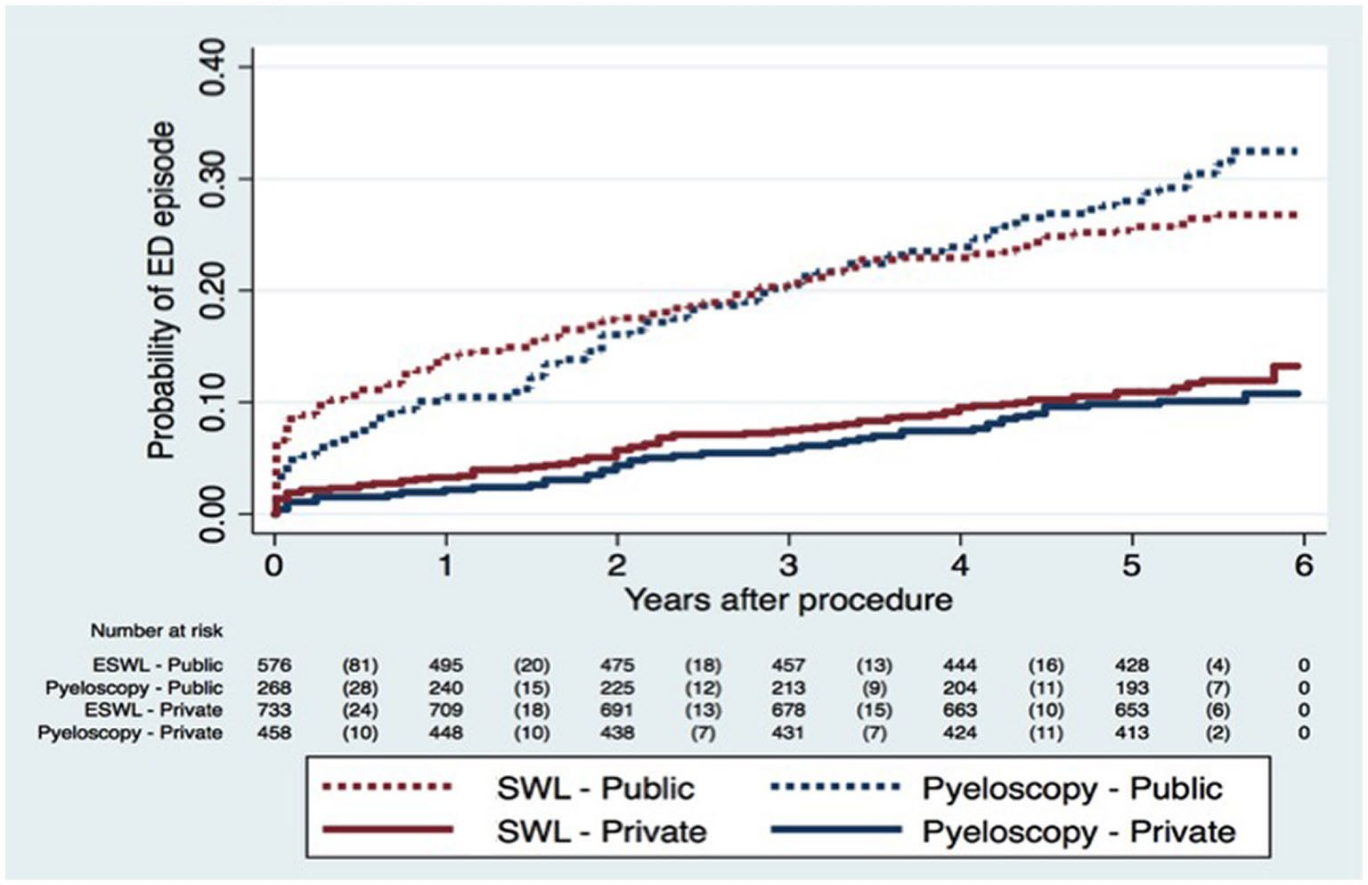
Probability of presentation to emergency department by procedure type and insurance status

We performed a multivariate analysis of the variables that were deemed clinically significant as well as those shown to be statistically significant/trended toward significance in our univariate analysis, in order to predict emergency presentations and unplanned re‐admissions. Regression included procedure type, stenting, age, insurance status, and length of stay. With respect to emergency presentations, only insurance status was a significant independent predictor, with public patients more likely to present to emergency within 12 months, (odds ratio [OR] 1.40, CI 1.04‐1.75, *P* < .001). This finding was durable over 6 years; regardless of the procedure type, patients treated initially in public were 2.17 times more likely to attend ED than those treated in private over the study period (*P* ≤ .001) (Figure 1). Both flexible URS ([OR] 1.67, CI 1.23‐2.26, *P* = .001) and being a public patient ([OR] 3.06, CI 2.24‐4.18, *P* < .001) significantly increased the likelihood that patients required an unplanned re‐admission within 12 months.

## 4 DISCUSSION

Our study compared patients with renal calculi treated by SWL and URS over a one‐year period, and with no urological stone surgery in the preceding 12 months. The endoscopic treatment of renal calculi has come to the forefront of urology both in Australia and globally, this has been due to multiple factors including increased training and advancements in scope and disposable technology.^11^, ^15^ Guidelines suggest that clinicians may offer SWL or URS as acceptable treatment options for non‐lower pole renal stone burdens less than 20 mm in size; where both these techniques have less morbidity than PCNL.^15^

The patients in this study had comparable clinical backgrounds and demographics; furthermore, both types of stone surgery were undertaken in a similar hospital setting in both public and private hospitals across Victoria, Australia. Our findings suggest that stone surgery in the public healthcare system confers a 40% increased risk of emergency presentation (*P* < .001) as well as 3.06 times the likelihood of an unplanned re‐admission at 12 months. We postulate that this finding is likely multi‐ factorial, with reasons for this including quality of patient education, analgesia plan, and easier direct contact with patients’ surgeon in the post‐operative period.

Our results show that the number of emergency presentations at 60 days was significantly higher for patients who had undergone SWL. However, at 12 months, flexible URS procedures accounted for a greater total proportion of emergency presentations, with these patients with complications presenting 3.00 times/patient, compared to 1.59 times/patient for SWL (*P* = .012). The most common complication encountered at emergency presentation was “renal colic pain.” In our cohort, ureteric stent insertion was significantly more prevalent in flexible URS patients (73% vs 3%), and even more so in flexible URS patients who presented to emergency post‐operatively (89%). However, having a stent did not confer significant added risk for emergency presentations or unplanned admission on multivariate analysis. In our cohort, stents were often inserted post‐endoscopic stone treatment; however, according to the AUA guidelines, stent insertion can be omitted if patients have all of the following: no evidence of ureteral injury or stricture, those with a normal contralateral kidney, normal kidney function, and nil further planned SWLs (Evidence Level Grade A).^15^ The high rate of ureteric stent insertion in flexible URS patients also accounted for the much higher rate of overall re‐admissions (including elective admissions for stent removal) in these patients, contributing significantly to the overall burden on the health care system, compared to SWL.

When we excluded elective re‐admissions and analyzed unplanned re‐admissions, flexible URS patients accounted for significantly more of these compared to SWL. The incidence of unplanned re‐admissions was 11.64% for flexible URS versus 9.03% for SWL (*P* = .036), and on multivariate analysis flexible URS conferred a 67% increased risk of unplanned re‐admission (*P* = .001). This unplanned re‐admission rate and higher complication rate associated with flexible URS compared to SWL is comparable to that reported in the literature.^16^ Furthermore, 9.74% of flexible URS patients required an overnight stay compared to (7.00% of SWL patients *P* = .029), which is also suggestive of the greater complexity and morbidity associated with flexible URS.^16^

## 5 LIMITATIONS

The limitations of our study include its retrospective nature and lack of randomization. Hospital diagnostic and billing codes were used to generate the data and complications, so while complication events could be under‐reported; we believe they were reported equally in both cohorts, eliminating potential biases of surgeon or patient‐reported outcomes. Furthermore, coding data were crosschecked and filtered to exclude any discrepancies and duplicates; however, accuracy is dependent on each hospitals’ coding. Due to the nature of the database used, stone characteristics such as location and size as well as specific operative details such as procedure time were not available for analysis. In this study, we focused on short‐ term complications and did not analyze stone‐free rates, we will look at 5 year stone outcomes for this cohort in a separate study.

## 6 CONCLUSION

In Victoria, patients with a renal calculus undergoing SWL were significantly more likely to present to emergency within 60 days; however, at 12 months, flexible URS patients surpassed SWL patients with respect to the number of emergency presentations. Furthermore, flexible URS patients were more likely to require an admission, both electively as well as unplanned.

Flexible URS was associated with more frequent and more serious complications in this population than SWL; additionally, the overall direct burden on the healthcare system in terms of time spent in hospital in a 12 months period per procedure was greater. This may partly represent more complicated calculi treated with flexible URS but should be taken into consideration for renal calculi with which both procedures are indicated.

Furthermore, our findings indicate that work is needed to reduce emergency presentations related to both SWL and flexible URS, symptoms at emergency presentation indicate that better patient education regarding stent management may be required, especially in the public health care system. In addition, the rate of ureteric stent placement is higher than expected based on AUA guidelines; avoidance of ureteric stent insertion when not indicated may reduce unplanned presentations post‐renal calculi procedures.

## CONFLICT OF INTEREST

Gregory Jack, Niall Davis, Matthew Farag, Lih‐Ming Wong: None.

Damien Bolton: Boston Scientific, Investigator Grant.

## References

[1] Türk C , Neisius A , Petřík A , Seitz C , Thomas K , Skolarikos A . EAU guidelines on urolithiasis [online guideline]. Arnhem, The Netherlands: EAU Guidelines Office; 2018 [cited 2019 Sep 9].

[2] Fisang C , Anding R , Müller SC , Latz S , Laube N . Urolithiasis–an interdisciplinary diagnostic, therapeutic and secondary preventive challenge. Dtsch Arztebl Int. 2015;112(6):83–91. 25721435 10.3238/arztebl.2015.0083 PMC4349965

[3] Hesse A , Brandle E , Wilbert D , Kohrmann KU , Alken P . Study on the prevalence and incidence of urolithiasis in Germany comparing the years 1979 vs. 2000. Eur Urol. 2003;44(6):709–13. 14644124 10.1016/s0302-2838(03)00415-9

[4] Curhan GC . Epidemiology of stone disease. Urol Clin North Am. 2007;34(3):287–93. 17678980 10.1016/j.ucl.2007.04.003 PMC2693870

[5] Stamatelou KK , Francis ME , Jones CA , Nyberg LM , Curhan GC . Time trends in reported prevalence of kidney calculi in the United States: 1976–1994. Kidney Int. 2003;63(5):1817–23. 12675858 10.1046/j.1523-1755.2003.00917.x

[6] Sanchez‐Martin FM , Millan Rodriguez F , Esquena Fernandez S , Segarra Tomas J , Rousaud Baron F , Martinez‐Rodriguez R , et al. Incidence and prevalence of published studies about urolithiasis in Spain. A review. Actas Urol Esp. 2007;31(5):511–20. 17711170 10.1016/s0210-4806(07)73675-6

[7] Asplin JR , Favus MJ , Coe FL . Nephrolithiasis. In: Brenner BM , editor. Brenner and rector's the kidney. 5th ed. Philadelphia: Saunders; 1996. p. 1893–935.

[8] Resnick MI , Persky L . Summary of the National Institutes of Arthritis, Diabetes, Digestive and Kidney Diseases conference on urolithiasis: state of the art and future research needs. J Urol. 1995;153(1):4–9. 7966786 10.1097/00005392-199501000-00004

[9] Clark JY , Thompson IM , Optenberg SA . Economic impact of urolithiasis in the United States. J Urol. 1995;154(6):2020–4. 7500448

[10] Holman CDJ , Wisniewski ZS , Semmens JB , Bass AJ . Changing treatments for primary urolithiasis: impact on services and renal preservation in 16 679 patients in Western Australia. BJU Int. 2002;90(1):7–15. 10.1046/j.1464-410x.2002.02804.x 12081761

[11] Perera M , Papa N , Kinnear N , Wetherell D , Lawrentschuk N , Webb D , et al. Urolithiasis treatment in Australia: the age of ureteroscopic intervention. J Endourol. 2016;30(11):1194–9. 27629239 10.1089/end.2016.0513

[12] Segura JW , Preminger GM , Assimos DG , Dretler SP , Kahn RI , Lingeman JE , et al. Ureteral calculi clinical guidelines panel summary report on the management of ureteral calculi. The American Urological Association. J Urol. 1997;158(5):1915–21. 9334635 10.1016/s0022-5347(01)64173-9

[13] Assimos D , Krambeck A , Miller NL , Monga M , Murad MH , Nelson CP , et al. Surgical management of stones: American Urological Association/endourological society guideline [online guideline]. UA/Endourological Society Guideline; 2016. [cited 2019 Sep 19].

[14] Parmar MS . Kidney calculi. BMJ. 2004;328(7453):1420. 15191979 10.1136/bmj.328.7453.1420 PMC421787

[15] Shah O . Surgical management of renal calculi: AUA/endourological society guideline. AUANews. 2016;21(5):4–6.

[16] Drake T , Grivas N , Dabestani S , Knoll T , Lam T , Maclennan S , et al. What are the benefits and harms of URS compared with shock‐wave lithotripsy in the treatment of upper ureteral calculi? A systematic review. Eur Urol. 2017;72(5):772–86. 28456350 10.1016/j.eururo.2017.04.016

